# Supplementary material for: Transcriptome Profiling and Metagenomic Analysis Help to Elucidate Interactions in an Inflammation-Associated Cancer Mouse Model
Source: Cancers (Basel). 2021 Jul 22;13(15):3683. doi: 10.3390/cancers13153683 (PMC8345192; doi:10.3390/cancers13153683)
Supplement: Supplementary file 1 [file cancers-13-03683-s001.zip › cancers-1218983-supplementary.pdf]

Fig. S1

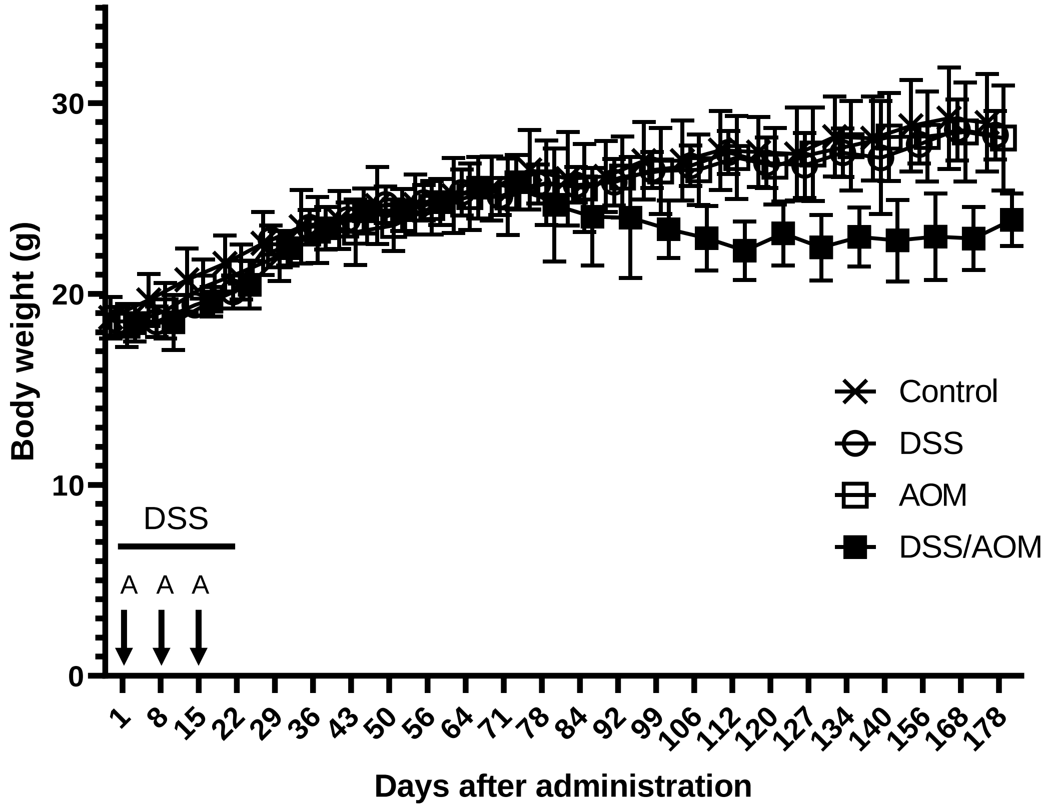

Figure S1. Body weight change among each group. The data were plotted until day 178 at the sacrifice. The lines represent mean ± SD.
A; AOM
